# The Corrosion Properties of Al-Cu-Mg Alloy Fabricated via Additive Friction Stir Deposition

**DOI:** 10.3390/ma19214630

**Published:** 2026-10-31

**Authors:** Hu Wang, Zhixiong Zhu, Yongyong Lin, Dawei Guo, Guoshun Yang, Chen Wang, Jinbao Chen

**Affiliations:** 1 School of Astronautics, Nanjing University of Aeronautics and Astronautics, Nanjing 210016, China; wanghu@aeespace.com (H.W.); nuaawangchen@nuaa.edu.cn (C.W.); chenjbao@nuaa.edu.cn (J.C.); 2 Aerospace Engineering Equipment (Suzhou) Co. Ltd., Suzhou 215104, China; linyongyong@aeespace.com (Y.L.); yangguoshun@aeespace.com (G.Y.); 3 Institute for Development & Quality, Macau, China; dwguo@idq.org.mo

**Keywords:** additive friction stir deposition, Al-Cu-Mg alloy, microstructure, corrosion performance

## Abstract

Using as-received Al-Cu-Mg bars as the raw material, a 530 mm diameter Al-Cu-Mg alloy ring component was successfully fabricated via additive friction stir deposition (AFSD). Owing to the severe plastic deformation and dynamic recrystallization induced during the AFSD process, the microstructure was noticeably refined. The average grain size decreased from 35.84 μm to approximately 3.40 μm, with over 90% of grains below 4 μm. Electrochemical impedance spectroscopy (EIS) and potentiodynamic polarization tests in 3.5 wt.% NaCl solution revealed that the AFSD-ed alloy exhibited a higher corrosion potential of −675.29 ± 7.78 mV (vs. Saturated Calomel Electrode, SCE) and a lower corrosion current density of 1.45 ± 0.04 × 10^−6^ A/cm^2^, compared with −684.33 ± 0.86 mV and 4.33 ± 1.30 × 10^−6^ A/cm^2^ for the as-received bar, respectively. EIS further confirmed superior corrosion resistance, as evidenced by larger capacitive arcs and a higher low-frequency impedance modulus. Microstructural characterization indicated that the corrosion mode, from propagating pits to isolated pits, is probably due to the refined grains and secondary phase particles during AFSD. These findings demonstrate that AFSD enables large, fully dense Al-Cu-Mg components with enhanced corrosion resistance, establishing AFSD as a promising route to improved corrosion performance for aerospace and defense applications.

## 1. Introduction

Al-Cu-Mg alloys stand as a group of critical materials in modern industry. Al-Cu-Mg alloys are critical materials in modern aerospace and defense industries due to their high specific strength and thermal resistance [1,2,3,4,5]. Recent advances in friction stir welding (FSW), as an innovative solid-state joining technology, have significantly improved their weldability, eliminating defects such as solidification cracking and porosity [6,7]. This success highlights the unique advantages of solid-state processing for Al-Cu-Mg alloys. Recently, additive friction stir deposition (AFSD), a solid-state additive technique derived from FSW, has emerged as a promising technology for component fabrication without melting.

From the design perspective, additive manufacturing (AM) promotes integrated near-net-shape forming. Thus, with the further application of limited subtractive manufacturing via computer numerical control (CNC) milling, AM will be the prevailing method for the fabrication of components with high comprehensive performance [8]. AFSD is a solid-state additive manufacturing technology that is highly suitable for repairing, joining, or creating fully dense materials with tailored microstructures [9]. The materials do not involve melting or solidification during the AFSD process.

During the AFSD process, friction-generated heat softens the substrate material through mechanical friction between the tool and workpiece. Subsequently, the tool induces mechanical stirring in the deposited layer, facilitating metallurgical bonding between successive layers. This layered accumulation mechanism enables the fabrication of three-dimensional components. The severe plastic deformation inherent in AFSD can generate a refined microstructure with equiaxed grains, often accompanied by good consolidation and well-bonded interfaces under optimized processing conditions. The mechanical properties of AFSD-ed parts are generally superior to traditional castings and can be comparable to those of forged materials, depending on alloy composition, feedstock condition, and processing parameters. AFSD process with characteristics of non-melting facilitates rapid production of near-net-shape components high-performance [10].

Compared with conventional FSW, AFSD offers the advantages of solid-state processing and near-net-shape fabrication, but its outcome depends strongly on processing parameters and thermal cycling. In this work, a ring-shaped component was selected because large ring structures are representative of aerospace and defense applications.

So far, several researchers have conducted investigations on the microstructure and mechanical properties of AFSD-ed Al-Cu-Mg alloys. For instance, Ghadimi et al. [11] found that the Al2050 (Al-Cu-Li-Mg-Zr) achieves a uniform hardness distribution after AFSD processing, with interlayer hardness differences less than 5 HV. Feng et al. [12] found that Al-Cu-Mg-Mg alloys processed by AFSD have a very fine recrystallized microstructure and excellent mechanical properties. The average grain sizes at the top, center, and bottom regions of the components are 3.0 μm, 4.7 μm and 4.8 μm, respectively. The tensile strength is 532 MPa with elongations of 31.2%. Griffiths et al. [13] examined the microstructural evolution during AFSD in Al-Cu-Mg, Al-Mg-Si-Cu and other alloys, providing a framework for subsequent research.

Recent studies on AFSD-processed 2xxx alloys have shown that grain refinement and intermetallic particle evolution can have competing effects on corrosion behavior [14,15]. Although these studies provide valuable insights into the corrosion response of AFSD-processed aluminum alloys, the corrosion behavior of AFSD-processed Al-Cu-Mg alloys remains largely unexplored. As mentioned earlier, corrosion resistance is a critical property of Al-Cu-Mg alloys [16,17], which significantly restricts their application. Therefore, it is imperative to conduct in-depth investigations on the corrosion behavior of AFSD-processed Al-Cu-Mg alloy components, so as to clarify the influence mechanisms of the AFSD process on the corrosion resistance of this alloy. This will lay a foundation for the future application of AFSD components of Al-Cu-Mg alloy.

In this study, an Al-Cu-Mg alloy ring with a diameter of 530 mm was fabricated using the AFSD process. The microstructure and corrosion properties of the resulting component were subsequently characterized to elucidate the influence mechanism of AFSD on the corrosion resistance of the Al-Cu-Mg alloy.

## 2. Materials and Methods

**Figure 1:**
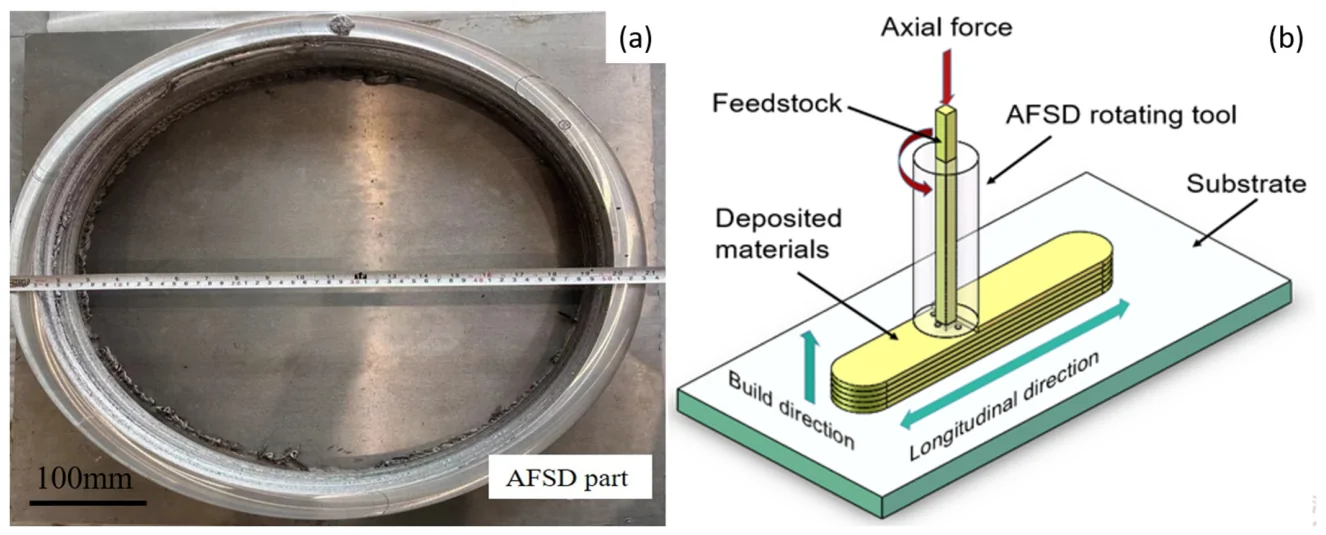
(**a**) Photo of as-deposited part; (**b**) schematic of AFSD process.

The deposited aluminum alloy was fabricated using solid-state manufacturing equipment (self-developed by Aerospace Engineering Equipment (Suzhou) Co., Ltd., Suzhou, China). Using the self-developed AFSD equipment, a circular ring-shaped part with a diameter of approximately 530 mm and a height of approximately 80 mm was fabricated on an aluminum alloy plate. During the AFSD process, the rotation speed was set at 450 rpm, while the traverse speed and reverse traverse speed were maintained at 150 mm/min. The feed rate was 20.91 mm/min. The tool was a double-scroll shoulder with a shoulder diameter of 28 mm and a filler outlet diameter of 15 mm. The substrate was an Al-Cu-Mg alloy plate with a thickness of 10 mm, the same composition as the feedstock rod. Deposition was performed layer-by-layer with a layer thickness of approximately 1.6 mm and a total height of approximately 50 mm. Here, “reverse traverse speed” refers to the tool travel speed when moving in the opposite direction during the reciprocating deposition strategy. A photograph of the as-deposited part is shown in Figure 1. The raw material used was an as-received Al-Cu-Mg alloy rod with a diameter of 15 mm and a length of 1000 mm. The chemical composition of the rod material is listed in Table 1.

The specimens for microstructural analysis were sectioned from the as-deposited part. The specimens were ground with SiC sandpaper and polished until no scratches remained on the surface. Among them, the metallographic specimens were mechanically polished with SiC sandpaper up to 2000 grit and then etched with 5% HF for 10 s. The macroscopic metallography of the cross-sections was examined using an optical microscope (OM, BX53M, Olympus, Tokyo, Japan) to confirm the presence of internal defects such as pores or weak bonding. The other specimens were subjected to electrolytic polishing in an electrolytic polishing solution (composed of 10% perchloric acid (HClO_4_) and 90% ethanol (CH_3_CH_2_OH)) to remove the mechanically disturbed surface layer and provide a suitable surface for EBSD analysis. Afterwards, the specimens were immersed in an alcohol solution to clean the surface and remove residual acid and polishing by-products. The electrolytic polishing was conducted at a voltage of 25 V, with a polishing duration ranging from 25 s to 40 s. The electrolytically polished specimens were then analyzed using electron backscatter diffraction (EBSD) in a scanning electron microscope (SEM, Gemini 500, Zeiss, Oberkochen, Germany) to obtain grain size, texture components, and grain boundary statistics. The EBSD data were collected at an accelerating voltage of 20 kV with a working distance of 15 mm and a step size of 0.5 μm. The mapped area was approximately 200 μm × 200 μm for the AFSD-ed sample and 1 mm × 1 mm for the as-received rod. One representative field was analyzed for each condition. The indexing rate was above 95%. A standard cleaning procedure was applied to remove wild spikes and non-indexed points. Grains were defined by a misorientation threshold of 15°, and low-angle grain boundaries were defined as misorientation angles below 15°.

Specimens with dimensions of 10 mm × 10 mm × 5 mm were cut from the central region of the additive manufactured ring and from the bar stock using wire electrical discharge machining (WEDM). A surface with an area of 10 mm × 10 mm was selected as the working surface, while the remaining surfaces were sealed with self-curing denture base resin. The working surface was ground with sandpaper of up to 2000 grit, followed by degreasing with alcohol and acetone, rinsing with deionized water, and drying. Subsequently, electrochemical tests were conducted in a 3.5 wt.% NaCl aqueous solution at 25 °C.

A three-electrode system was employed for the tests, where the counter electrode was a platinum electrode, the reference electrode was a saturated calomel electrode (SCE), and the working electrode was the aluminum alloy specimen under test. For the polarization curves, the scanning range was set from −0.90 V to +0.25 V (vs. SCE) with a scanning rate of 1 mV/s. The Tafel slopes (*β_a_* and *β_c_*) and corrosion current density (*I_corr_*) were determined by Tafel extrapolation using the CHI760e electrochemical workstation (Shanghai Chenhua, China). The potential intervals selected for the Tafel fitting were within *E_corr_* ± 100 mV for both the anodic and cathodic branches, where the linear Tafel regions were clearly identified. The fitting quality was evaluated by the linear regression coefficient, and the R^2^ values were greater than 0.98 for all fitted branches. The relatively low cathodic slopes are likely related to the specific surface condition of the Al-Cu-Mg alloy in the 3.5 wt.% NaCl solution and to the mixed activation–diffusion control of the cathodic process. Electrochemical impedance spectroscopy (EIS) measurements were performed at open circuit potential (OCP) with a sinusoidal perturbation amplitude of 10 mV and a scanning frequency range of 0.01 Hz to 100,000 Hz. To ensure the stability and accuracy of the test data, the electrochemical performance tests for both the raw material rod and the AFSD-ed specimens were conducted using two pieces, and the average of the measured values was taken as the basis for evaluating the electrochemical performance. The electrochemical performance testing primarily involved measuring the open circuit potential (OCP) of the specimens. The OCP was considered stable when the potential variation was less than 1 mV/min over the 1800–3600 s interval. To avoid the irreversible modification of the surface by polarization, EIS and potentiodynamic polarization measurements were performed on independent specimens. The equivalent circuit fitting was performed using ZView software 3.1 with the weighted least squares method.

Meanwhile, additional specimens were separately cut from the as-deposited part and the solid bar via WEDM for scanning electrochemical microscopy (SECM) analysis in the 3.5 wt.% NaCl solution. SECM analysis was performed using a scanning electrochemical microscope (SECM, TN, USA) with a Pt microelectrode of 10 μm diameter. The measurements were carried out in the feedback mode at a probe potential of −0.5 V (vs. SCE) and a tip-to-sample distance of 10 μm. The scan rate was 10 μm/s, and the spatial step was 2 μm. The scanning area was 400 μm × 400 μm. No mediator was used. The tip current was recorded in nA. One representative region was analyzed for each sample. After the corrosion tests, the surface of the specimens was analyzed using a scanning electron microscope (SEM, Gemini 500, Zeiss, Oberkochen, Germany).

## 3. Results

### 3.1. Microstructure

**Figure 2:**
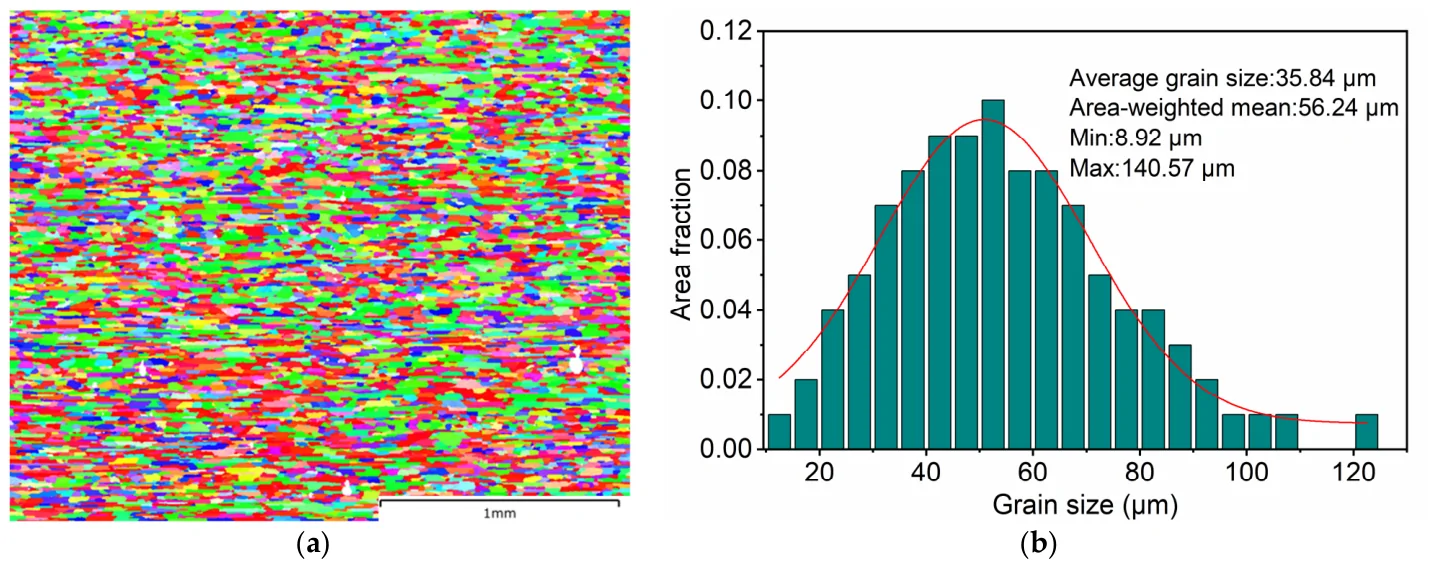
EBSD analysis of Al-Cu-Mg alloy rod (cross-section): (**a**) inverse pole figure; (**b**) grain size distribution. The “average grain size” is the number-averaged value, while the “area-weighted mean” is a separate statistical descriptor. The IPF map uses the standard color coding for the [001], [101], and [111] directions, with the rolling direction (RD) as the reference direction.

**Figure 3:**
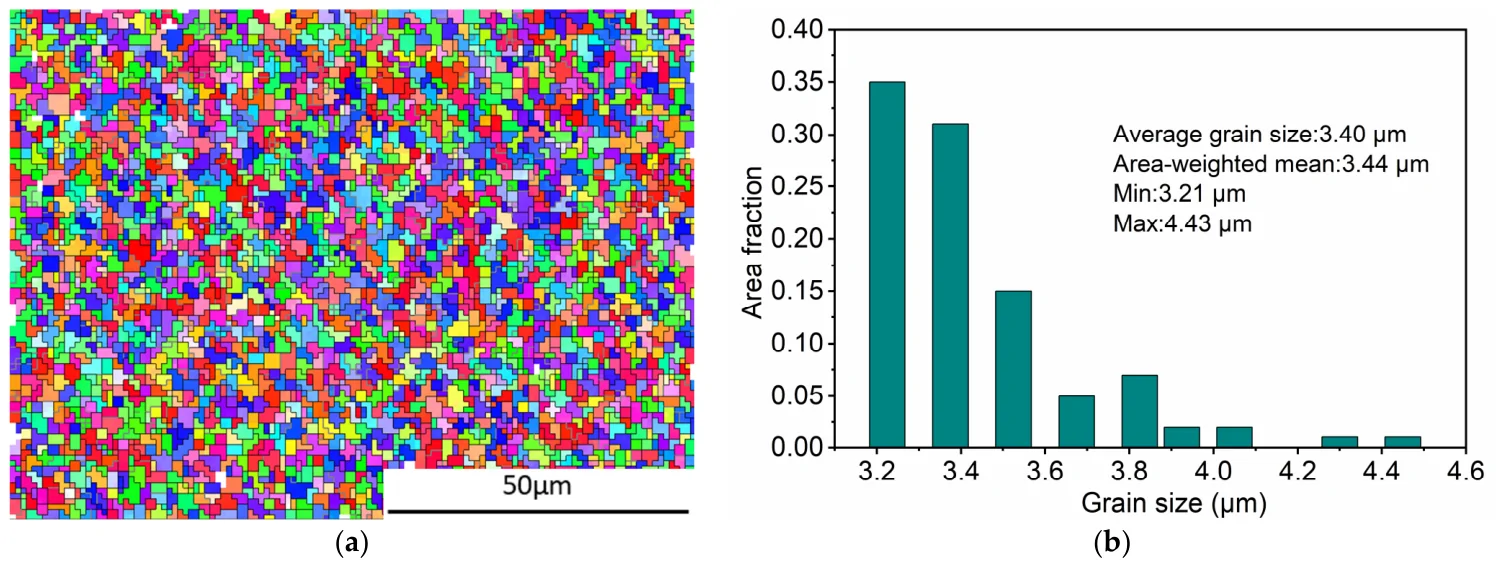

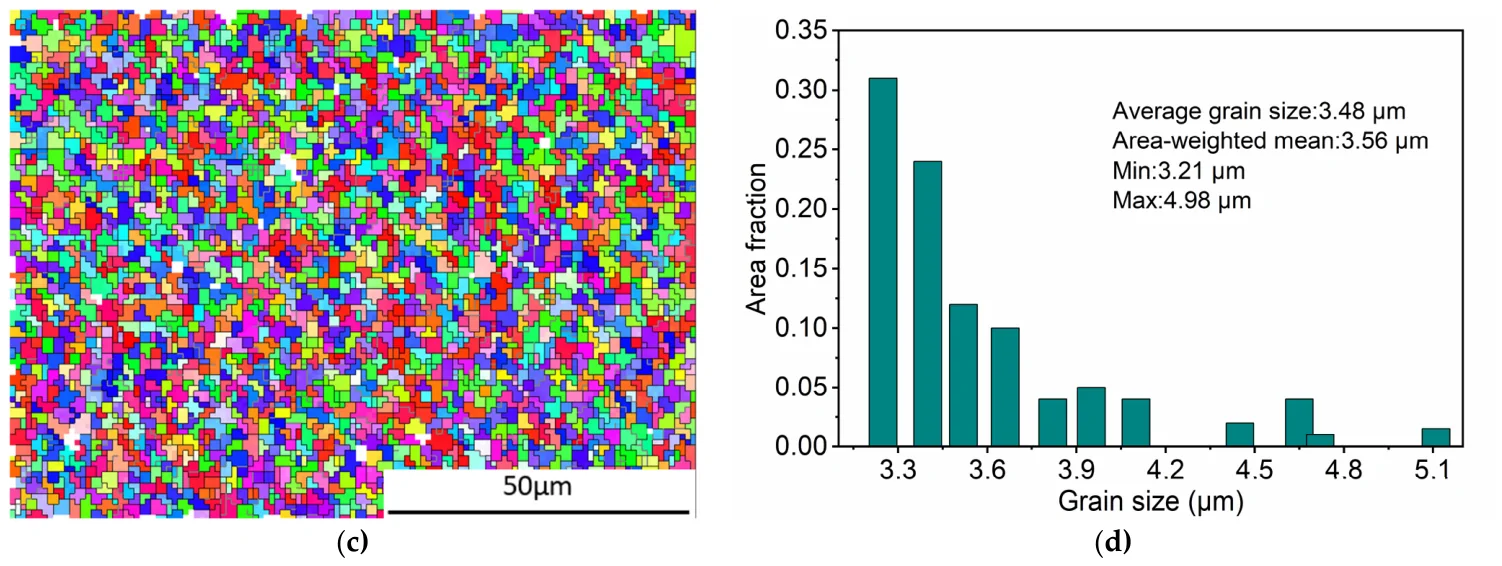
EBSD analysis of AFSD-ed Al-Cu-Mg alloy: (**a**) inverse pole figure in central region; (**b**) grain size distribution in central region; (**c**) inverse pole figure in bottom region; (**d**) grain size distribution in bottom region. The “average grain size” is the number-averaged value, while the “area-weighted mean” is a separate statistical descriptor. The IPF map uses the standard color coding for the [001], [101], and [111] directions, with the building direction (BD) as the reference direction.

EBSD characterization was conducted on the as-received Al-Cu-Mg alloy rod and the AFSD-ed sample. Inverse pole figure (IPF) maps and grain size statistics are shown in Figure 2 and Figure 3.

The as-received rod exhibited a typical rolled microstructure with elongated grains and an average grain size of 35.84 μm (Figure 2a,b). In contrast, the AFSD-ed sample displayed a refined equiaxed grain structure. The average grain size was 3.40 μm in the central region (Figure 3a,b) and 3.48 μm in the bottom region (Figure 3c,d). Grain size distribution histograms revealed that over 90% of the area fraction in the as-deposited sample was controlled within 4.00 μm. No obvious preferred orientation was observed in either the as-received rod or the AFSD-ed sample based on the IPF maps.

**Figure 4:**
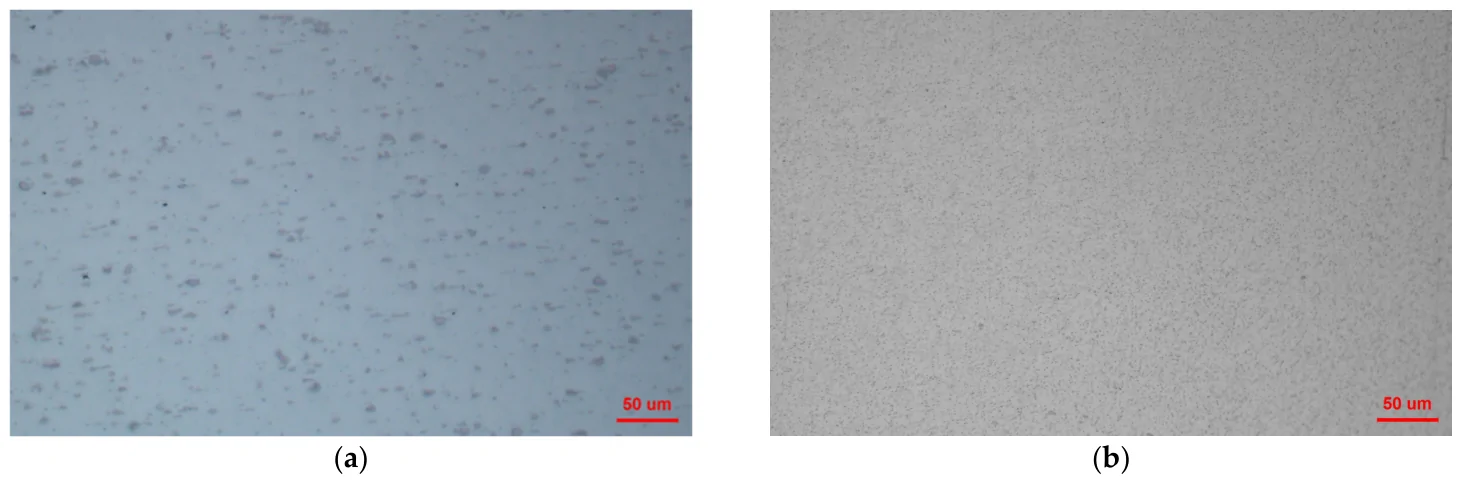
Microstructure showing the secondary phase morphologies before corrosion: (**a**) the as-received bar and (**b**) the AFSD-ed sample.

To further compare the secondary phase characteristics before the corrosion process, the microstructures of the as-received bar and the AFSD-ed sample were characterized. As shown in Figure 4, the as-received bar contains relatively large and unevenly distributed secondary phase particles. In contrast, the AFSD-ed sample exhibits significantly refined and more uniformly dispersed secondary phase particles. This microstructural refinement and homogenization are expected to reduce the formation of strong micro-galvanic couples in the alloy.

### 3.2. Electrochemical Performance

**Figure 5:**
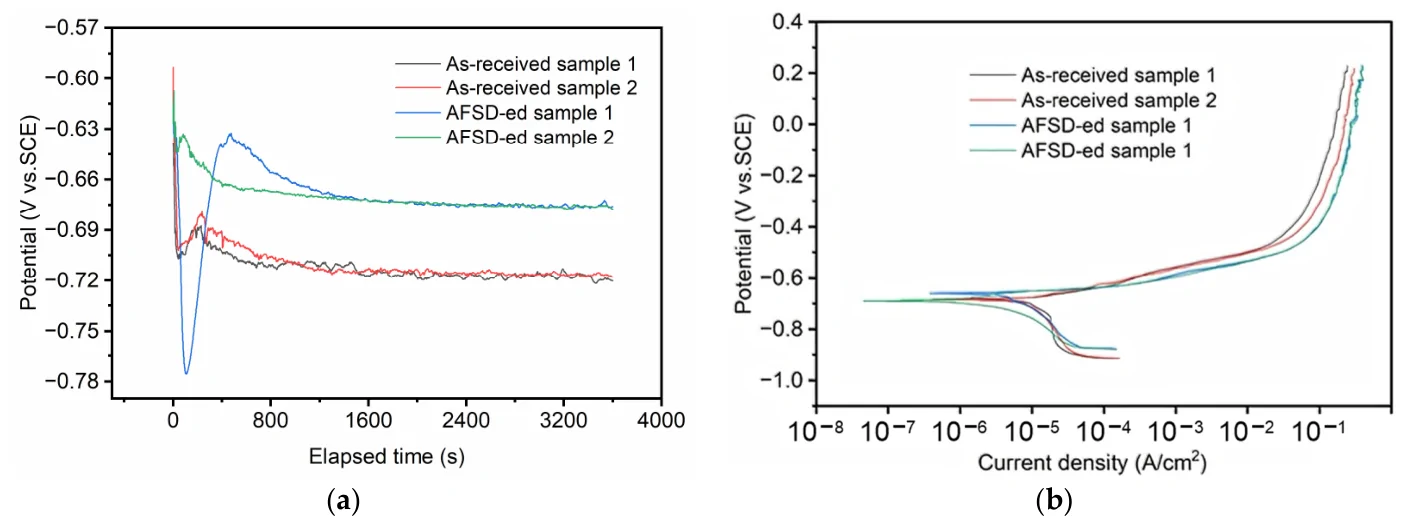
Electrochemical testing of AFSD-ed Al-Cu-Mg alloy and as-received rod: (**a**) OCP curves; (**b**) potentiodynamic polarization curves.

The open circuit potential (OCP) curves of the as-received rod and the AFSD-ed sample in 3.5 wt% NaCl solution are shown in Figure 5a. Both specimens exhibited initial fluctuations in OCP, which gradually stabilized with increasing immersion time (1800–3600 s). The stabilized OCP values were approximately –0.72 V (vs. SCE) for the as-received rod and –0.67 V (vs. SCE) for the AFSD-ed sample.

Potentiodynamic polarization curves are presented in Figure 5b, and the quantitative parameters are summarized in Table 2. The AFSD-ed sample exhibited a higher corrosion potential (*E_corr_* = –675.29 mV vs. SCE) compared to the as-received rod (*E_corr_* = –684.33 mV vs. SCE), representing a positive shift of approximately 9 mV. The corrosion current density (*I_corr_*) decreased from 4.33 μA/cm^2^ (as-received) to 1.45 μA/cm^2^ (AFSD-ed). The Tafel slopes *βa* and *βc* for the AFSD-ed samples were in the ranges of 0.013–0.024 V/dec and –0.0072 to –0.0077 V/dec, respectively, while those for the as-received rod were 0.029–0.034 V/dec and –0.0092 to –0.0096 V/dec.

### 3.3. Electrochemical Impedance Spectroscopy

**Figure 6:**
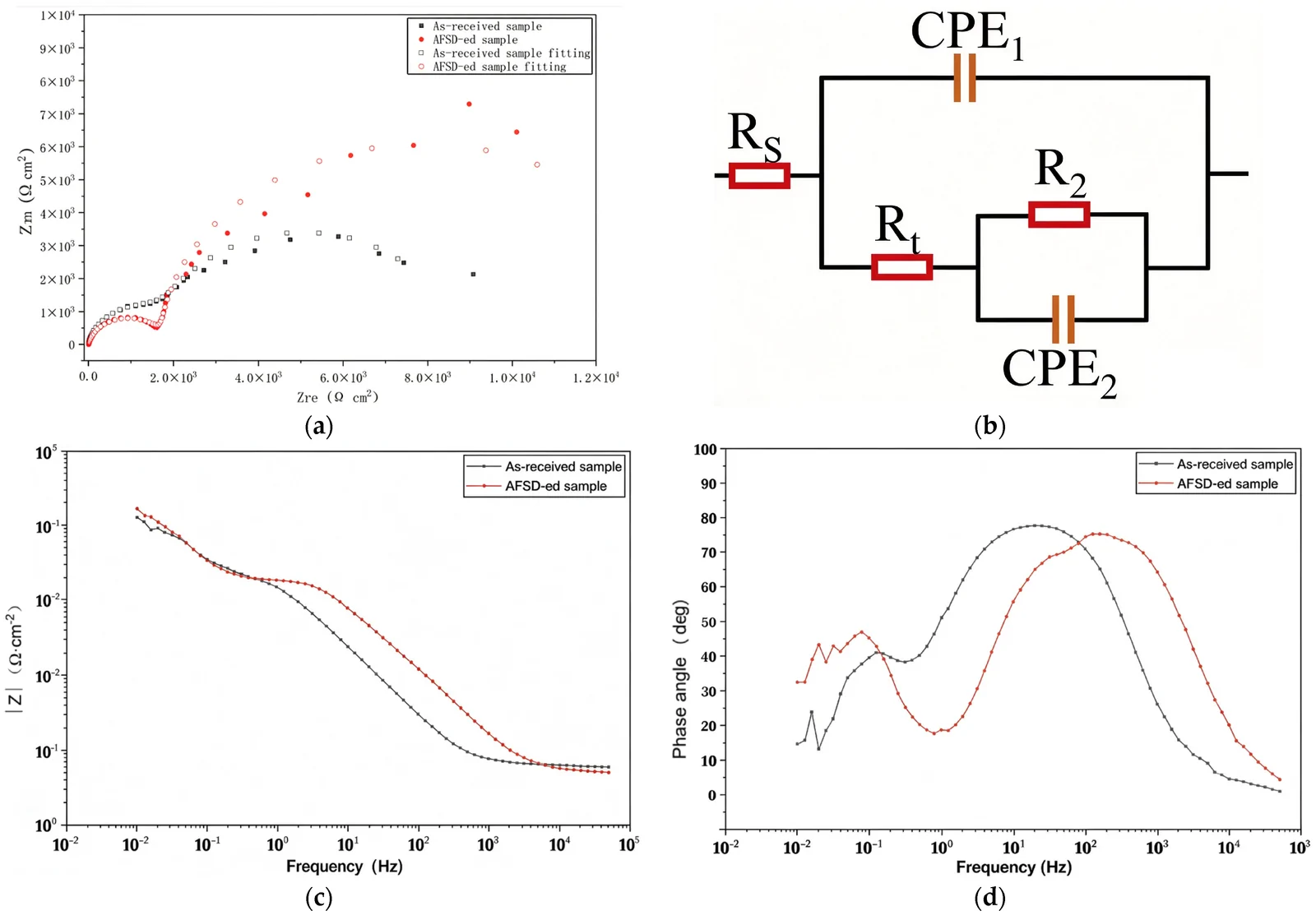
EIS analysis of the as-received rod and AFSD-ed Al-Cu-Mg alloy: (**a**) Nyquist curves; (**b**) equivalent circuit model; (**c**) impedance modulus (*|Z|*) versus frequency; (**d**) phase angle versus frequency.

Figure 6 presents the EIS results, including Nyquist plots, Bode plots, and the equivalent circuit model used for fitting. Both samples exhibited two distinct capacitive arcs in the Nyquist plots (Figure 6a). The AFSD-ed sample showed a larger capacitive arc radius compared to the as-received rod. The equivalent circuit model (Figure 6b) consisted of the solution resistance (*Rs*), a high-frequency loop representing the passive film response (*R_t_* and *CPE*_1_), and a medium-to-low-frequency loop representing the charge transfer process at the electrode/electrolyte interface (*R*_2_ and *CPE*_2_). This circuit was selected because both samples exhibit two distinct capacitive arcs in the Nyquist plots, and the two time constants are clearly resolved in the Bode phase angle plots. The high-frequency arc is associated with the surface passive film, while the medium-to-low-frequency arc is related to the charge transfer process at the electrode/electrolyte interface. The fitted parameters are listed in Table 3. In this model, the polarization resistance (*R_p_*) can be expressed by the following equation:

$$R_{P}=\frac{\beta_{a}\left|\beta_{c}\right|}{2.303i_{\mathrm{corr}}\left(\beta_{a}+\left|\beta_{c}\right|\right)}$$

where *β_a_* and *|β_c_|* are the absolute values of the anodic and cathodic Tafel slopes, respectively. The cathodic Tafel slope *β_c_* is reported as a negative value in Table 2 to indicate the cathodic polarization direction, but its absolute value is used in the Stern–Geary calculation.

After AFSD processing, the following changes were observed in the fitted parameters: *Rs* decreased from 6.271 to 5.236 Ω·cm^2^; the *CPE*_1_ parameter *Q* decreased from 8.969 × 10^−5^ S·s^n^·cm^−2^ to 2.364 × 10^−6^ S·s^n^·cm^−2^; *Rt* decreased from 2.588 × 10^3^ Ω·cm^2^ to 1.843 × 10^3^ Ω·cm^2^; and *R_2_* increased from 5.976 × 10^3^ Ω·cm^2^ to 1.227 × 10^4^ Ω·cm^2^. The χ^2^ values for both fittings are on the order of 10^−3^, indicating good agreement between the model and the experimental data. The total interfacial resistance (*R_t_* + *R_2_*) increases from approximately 8.56 × 10^3^ to 1.41 × 10^4^ Ω·cm^2^ after AFSD, indicating a higher overall interfacial impedance.

In the Bode plots (Figure 6c,d), the AFSD-ed sample exhibited higher impedance modulus *|Z|* values across the entire frequency range, particularly in the medium-to-low frequency range (<10^3^ Hz). The phase angle peak of the AFSD-ed specimen was broader and shifted toward lower frequencies compared to the as-received rod.

### 3.4. Corrosion Surface Morphology

**Figure 7:**
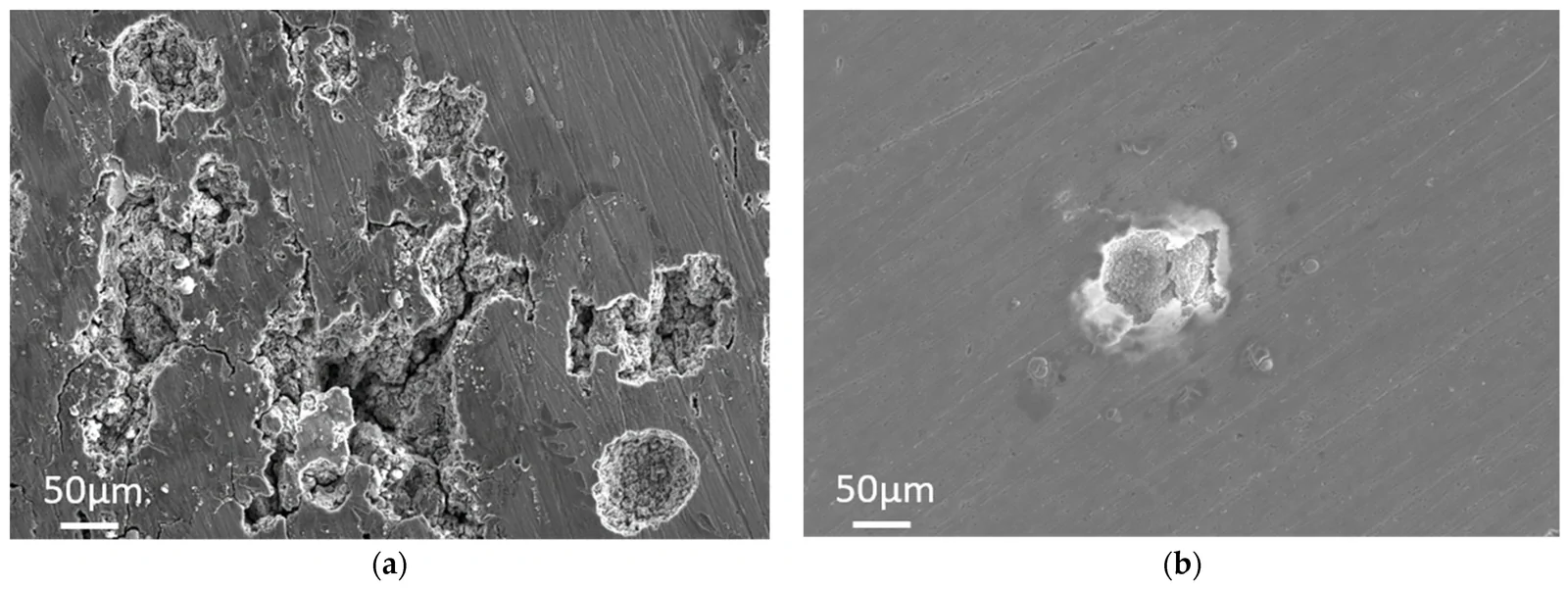
Corrosion morphology: (**a**) as-received Al-Cu-Mg rod; (**b**) AFSD-ed Al-Cu-Mg specimen.

The corrosion morphologies after immersion in 3.5 wt.% NaCl solution at 25 °C for 24 h are shown in Figure 7. The as-received rod (Figure 7a) displayed deep etch pits with rough and irregular edges, interconnected corrosion channels, and microcracks. The AFSD-ed sample (Figure 7b) exhibited only isolated pits with well-preserved surrounding matrix integrity.

**Figure 8:**
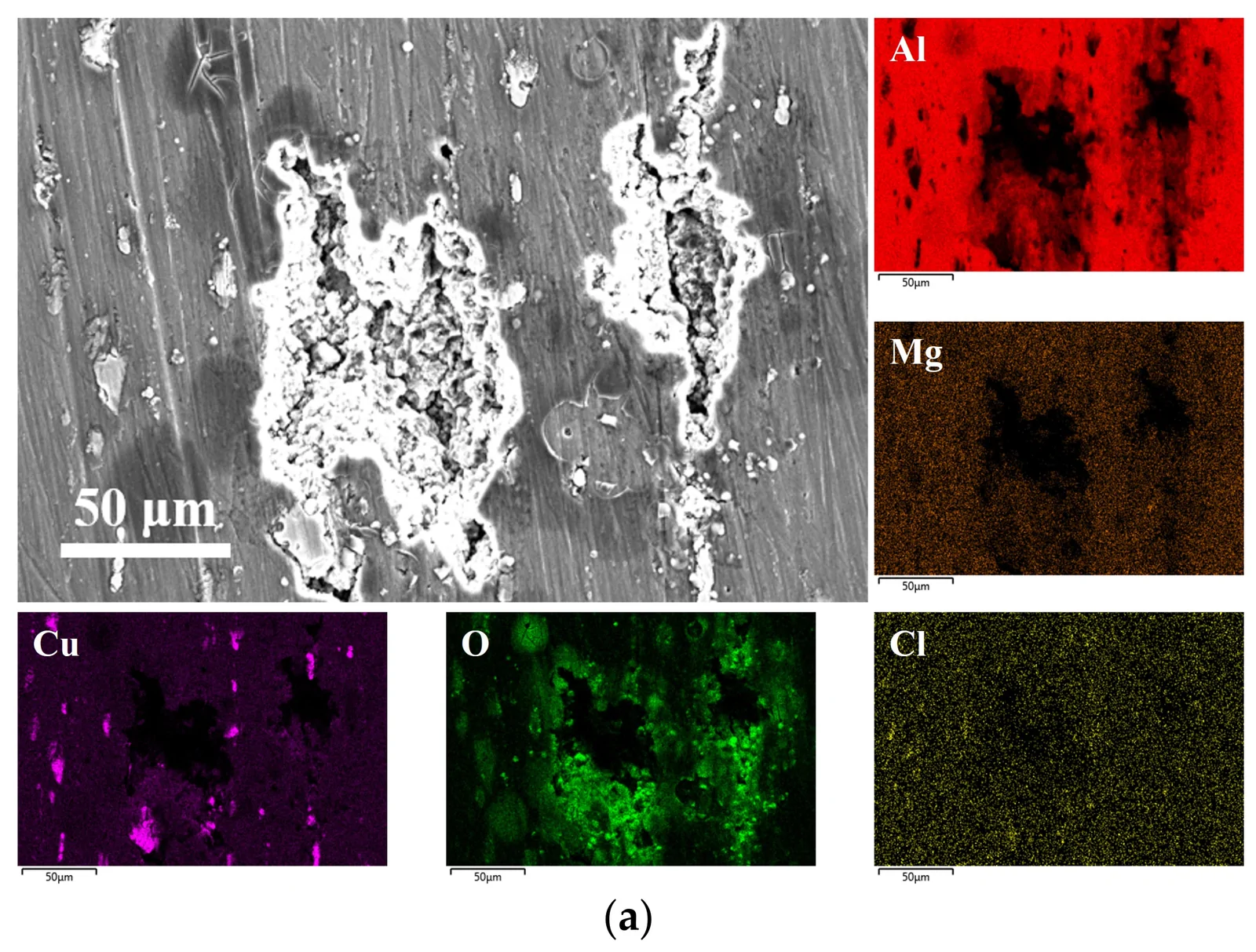

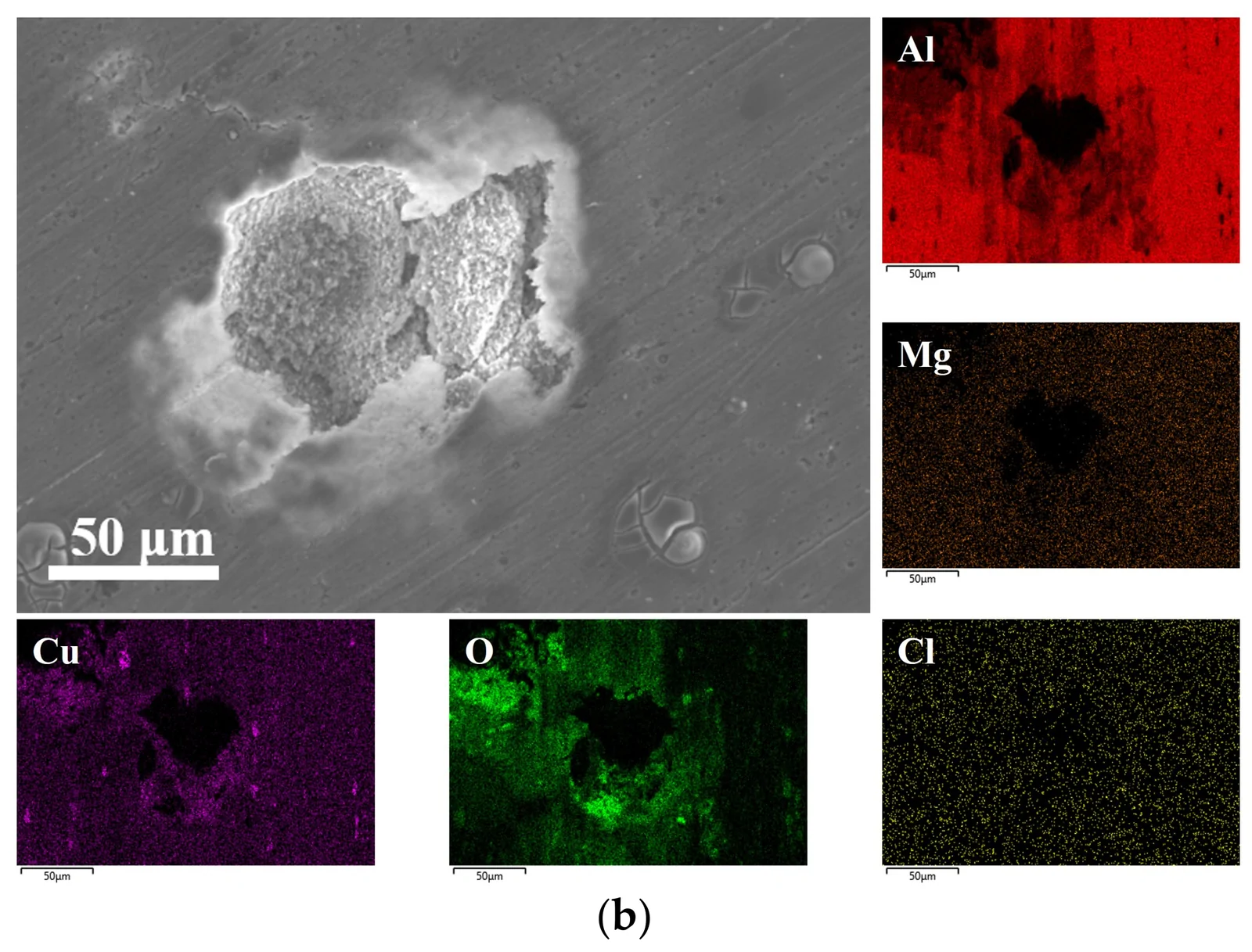
SEM morphology and corresponding EDS elemental maps (Al, Mg, Cu, O, Cl) of the corroded surfaces of (**a**) as-received bar and (**b**) AFSD-ed Al-Cu-Mg alloy.

**Figure 9:**
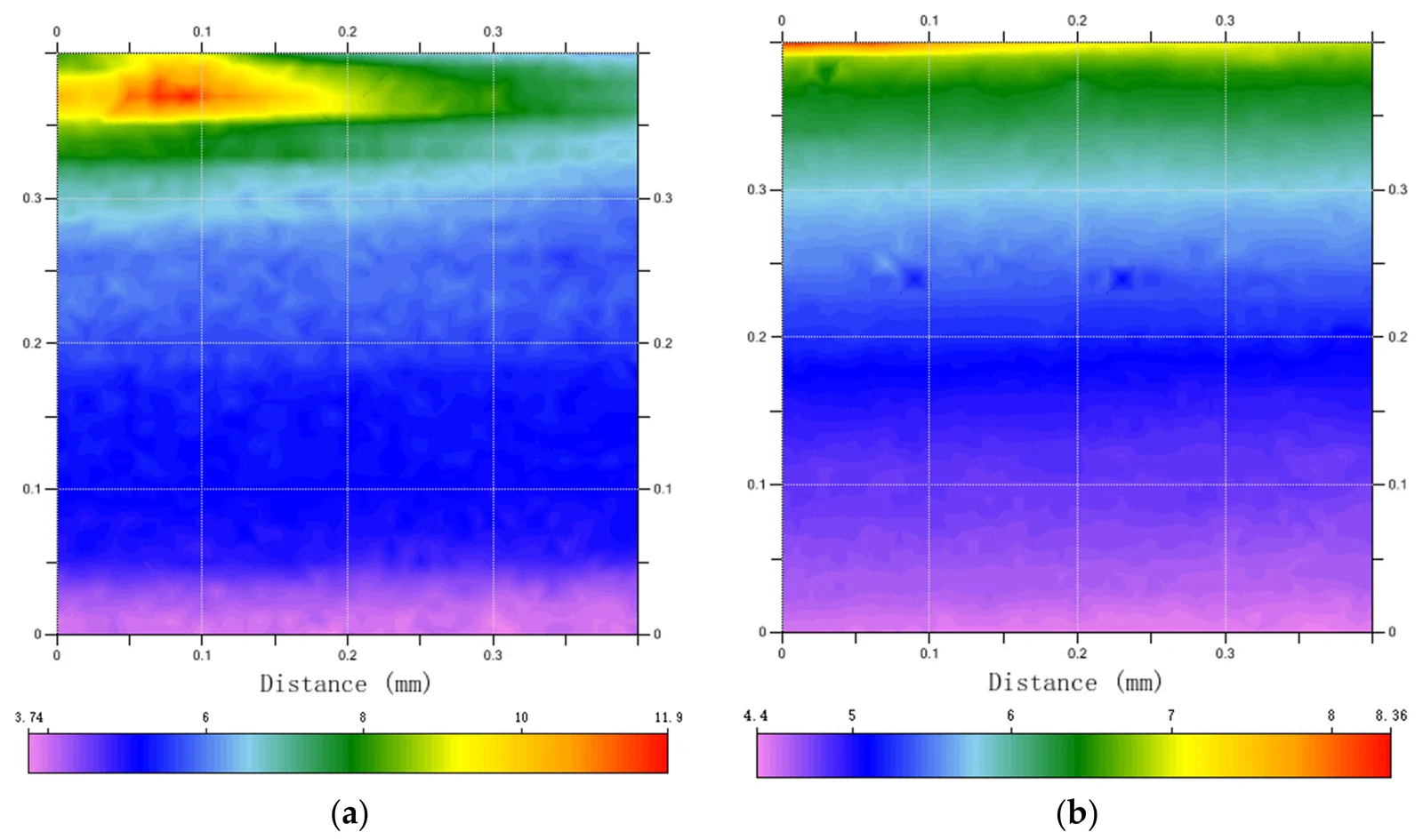
SECM analysis of Al-Cu-Mg alloy: (**a**) as-received bar; (**b**) AFSD-ed Al-Cu-Mg alloy. The color scale represents the tip current in nA.

EDS mapping of the corroded surfaces is presented in Figure 8. For the as-received rod (Figure 8a), the corroded region showed significant attenuation of the Al signal over a large area, localized Cu enrichment, and substantial O intrusion, along with a relatively strong and heterogeneous Cl signal. For the AFSD-ed sample (Figure 8b), the Al dissolution zone was narrower, the Cu distribution was more uniform with no obvious aggregation, and O enrichment was substantially reduced, with a much weaker Cl signal. SECM analysis (Figure 9) revealed that the as-received rod surface contained extensive high-activity zones (red and yellow regions) in the upper area, indicating concentrated anodic dissolution sites. The AFSD-ed sample (Figure 9b) showed narrowed and attenuated high-activity zones with reduced spatial extent and intensity.

## 4. Discussion

The significant grain refinement observed in the AFSD-ed sample (from 35.84 μm to ~3.40 μm) is primarily attributed to dynamic recrystallization (DRX) induced by severe plastic deformation during the AFSD process. The shoulder region of the AFSD tool exerts intensive extrusion and shear forces on the original rod material, promoting dynamic recrystallization and forming fine and uniform equiaxed grains.

The grain size distribution, with over 90% of grains below 4 μm, further confirms the grain refinement during AFSD. The grain boundaries in the AFSD-ed sample may include a relatively high fraction of low-angle boundaries, which are composed of organized dislocation structures. However, quantitative grain-boundary misorientation statistics were not obtained in this work.

The positive shift in both OCP (~50 mV) and *E_corr_* (~9 mV) after AFSD may be related to a change in the surface passive state, although this interpretation requires further verification. The decrease in *I_corr_* by approximately 66% indicates lower estimated corrosion kinetics under the adopted polarization conditions, but this trend should not be generalized to all corrosion regimes. The reduction in Tafel slopes (*β_a_* and *β_c_*) suggests that both anodic dissolution and cathodic reduction kinetics may be suppressed under the tested conditions, possibly due to the formation of a protective surface film.

Recent studies on AFSD-processed 2xxx alloys have shown that grain refinement and intermetallic particle evolution can have competing effects on corrosion behavior. In AFSD 2219, refinement of Al_2_Cu particles was accompanied by an increase in their number, leading to greater localized corrosion susceptibility [14]. In contrast, AFSD of AA2024 produced simultaneous grain refinement, fragmentation of coarse particles, *S*-phase coarsening, and changes in particle clustering, with complex effects on corrosion initiation and stable pitting [15]. In the present work, the AFSD-ed Al-Cu-Mg alloy exhibits significant grain refinement and a transition from deep interconnected pits to isolated shallow pits. The possible role of secondary-phase refinement and homogenization is discussed as a hypothesis rather than a directly established mechanism.

The improvement in corrosion resistance may be related to several factors, among which grain refinement is one contributing factor rather than an intrinsic benefit. As discussed above, the effect of grain size in Al-Cu-Mg alloys is coupled to dislocation density, precipitation state, constituent-particle distribution, solute segregation, and passive-film behavior [18,19,20].

Firstly, grain refinement increases the grain boundary density, which facilitates the rapid nucleation and growth of a dense, continuous passive film. High-density grain boundaries act as fast diffusion paths for alloying elements and vacancies, promoting uniform film formation. When the passive film is locally damaged, the surrounding fine-grained structure enables prompt re-passivation, effectively suppressing the propagation of pitting corrosion.

Secondly, microstructural homogenization may reduce compositional segregation at grain boundaries. In the as-received rod, segregated intermetallic phases create micro-galvanic couples with the α-Al matrix. After AFSD, the more uniform distribution of fine grains may help reduce local potential differences, thereby lowering the tendency for intergranular corrosion.

Thirdly, the presence of uniformly dispersed secondary phases is beneficial in reducing the local current density. This dispersion effect mitigates localized attack and promotes uniform corrosion behavior.

The EIS results provide further insight into the mechanisms of the improved corrosion resistance. The two capacitive arcs observed in both samples indicate a two-step corrosion process, which were a high-frequency response associated with the surface passive film and a medium-to-low-frequency response related to charge transfer across the electrode/electrolyte interface.

The significant increase in *R*_2_ (from 5.976 × 10^3^ to 1.227 × 10^4^ Ω·cm^2^) after AFSD demonstrates that the passive film becomes more resistant to ionic penetration and electronic transfer. This is consistent with the SEM/EDS observations showing reduced O intrusion and less extensive corrosion product accumulation. The decrease in the CPE1-Q value indicates a change in the capacitive response of the interface, which may be related to the formation of a more compact interfacial layer with fewer defects.

The increase in |Z| across the entire frequency range, especially in the medium-to-low frequency region, confirms that charge transfer is more difficult at the AFSD-ed surface. The broader phase angle peak and its shift toward lower frequencies indicate a wider distribution of relaxation times. This suggests a more distributed interfacial response, but it does not by itself demonstrate increased interfacial homogeneity. The decrease in n1 from 0.9309 to 0.8979 also indicates a greater deviation from ideal capacitive behavior, which should be considered when interpreting the interfacial properties. These EIS characteristics support the conclusion that AFSD transforms the electrode/electrolyte interface from a heterogeneous, highly active state to a homogeneous, passivation-dominated state.

In Al-Cu-Mg alloys, the *θ* (Al_2_Cu) and *S* (Al_2_CuMg) phases play critical roles in localized corrosion [12,14]. The *θ* phase is cathodic relative to the α-Al matrix, while the *S* phase is initially anodic. However, the *S* phase can undergo selective dissolution of Al and Mg, leaving a Cu-enriched remnant that subsequently acts as a local cathode and contributes to the dissolution of the surrounding α-Al matrix. In the as-received rod, these phases are coarse, unevenly distributed, and often agglomerated, creating strong galvanic couples. After AFSD, the refinement and more uniform distribution of these phases may weaken the micro-galvanic driving force and reduce the severity of localized corrosion. The narrow spacing between coarse cathodic phases leads to highly concentrated local current density, promoting severe pitting corrosion that propagates along phase boundaries to form deep, interconnected pits.

AFSD processing may refine both *θ* and *S* phases through severe plastic deformation and dynamic recrystallization. This may reduce localized enrichment of coarse cathodic phases and weaken the formation of strong micro-galvanic couples. With reduced galvanic driving force and dispersed anodic/cathodic sites, pitting initiation is limited to isolated micro-regions, resulting in shallow, non-propagating pits as observed in Figure 7b. However, it should be noted that quantitative pre-corrosion characterization of the θ and S phase size, area fraction, and spatial distribution was not performed in this work. Therefore, this interpretation should be considered a hypothesis rather than a directly established mechanism.

The EDS results further confirm this explanation. The corresponding anodic and cathodic reactions in 3.5 wt.% NaCl solution are: Al → Al^3+^ + 3e^−^ (anodic) and O_2_ + 2H_2_O + 4e^−^ → 4OH^−^ (cathodic). Based on the EDS results and the established corrosion behavior of Al-Cu-Mg alloys in chloride-containing solutions, the main corrosion products are expected to be Al(OH)_3_ and Al_2_O_3_. The severe Al depletion and Cu enrichment in the as-received rod confirm intensive galvanic corrosion around Cu-rich phases [15,21]. After AFSD, the uniform Cu distribution and reduced O enrichment indicate that galvanic coupling is effectively weakened and the anodic dissolution rate is suppressed [21,22,23].

SECM provides spatial mapping of electrochemical activity, complementing the morphological and compositional analyses. The extensive high-activity zones in the as-received rod may be associated with regions containing coarse secondary phases, which could act as local cathodes and promote anodic dissolution of the surrounding matrix. The narrowed and attenuated activity zones in the AFSD-ed sample are consistent with a more homogeneous microstructure containing finer and more dispersed secondary phases. The SECM high-activity zones and the corrosion pits observed in Figure 7a appear to be spatially related, but direct co-registration of SECM, SEM, and EDS data was not performed in this work. Therefore, this spatial relationship is discussed as a plausible interpretation rather than a directly demonstrated correlation.

**Figure 10:**
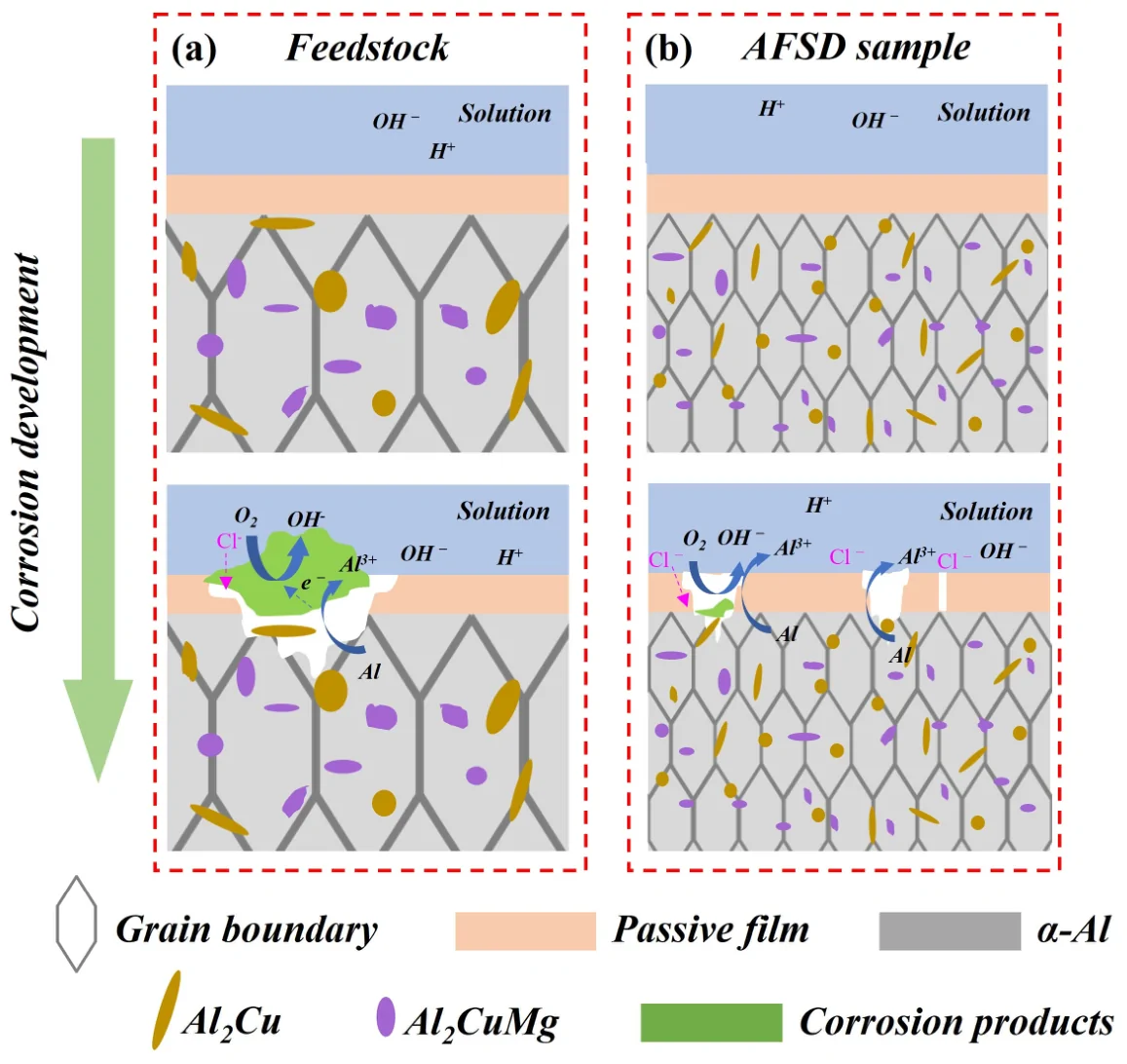
Schematic illustration of the corrosion behavior of (**a**) as-received and (**b**) AFSD-ed Al-Cu-Mg alloy before and after 24 h immersion in 3.5 wt.% NaCl solution. The as-received sample exhibits extensive pits and interconnected corrosion channels, whereas the AFSD-ed sample shows isolated pits. The green areas represent the porous corrosion products (mainly Al(OH)_3_, Al_2_O_3_, and AlCl_3_).

To better illustrate the corrosion process, a schematic diagram summarizing the corrosion behavior of the as-received and AFSD-ed Al-Cu-Mg alloy before and after 24 h immersion in 3.5 wt.% NaCl solution is presented in Figure 10. In this schematic, the passive film (blue layer) refers to the dense and protective native oxide layer (mainly Al_2_O_3_) on the alloy surface, which acts as a barrier against Cl^−^ ingress. In contrast, the green areas represent the accumulated corrosion products after the passive film breaks down. These products are a complex mixture mainly consisting of aluminum hydroxide (Al(OH)_3_), aluminum oxide (Al_2_O_3_), and aluminum chloride (AlCl_3_) [14]. Unlike the dense passive film, these corrosion products are porous and loose. They cannot prevent Cl^−^ penetration; instead, they hinder the diffusion of Al^3+^, causing localized enrichment of Al^3+^ and creating an autocatalytic effect that further accelerates localized corrosion.

Furthermore, the as-received sample exhibits coarse grains and unevenly distributed secondary phases, which promote strong micro-galvanic coupling between the *θ*-Al_2_Cu phase (cathode) and the α-Al matrix (anode) [24]. This leads to extensive anodic dissolution, resulting in interconnected corrosion pits and channels [21]. In contrast, the AFSD-ed sample exhibits significant grain refinement and a potentially more uniform distribution of secondary phases. This microstructural change may reduce the number of strong galvanic couples and disperse the local current density, thereby confining corrosion to isolated pits without interconnected channels.

In summary, the improved corrosion resistance of the AFSD-ed Al-Cu-Mg alloy is likely related to the combined effects of grain refinement, secondary phase redistribution, and passive film changes [25,26,27]. AFSD may transform the corrosion mode from synergistically propagating pitting to isolated, suppressed pitting. The contributing factors may include: (1) dynamic recrystallization-induced grain refinement that increases grain boundary density and may promote rapid passivation; (2) more uniform dispersion of fine secondary phases that may reduce micro-galvanic driving forces [28]; and (3) reduced compositional heterogeneity that may weaken preferential corrosion paths [29,30]. These findings highlight the potential of solid-state additive manufacturing to improve the corrosion behavior of Al-Cu-Mg alloys, while further quantitative characterization is needed to fully confirm the proposed mechanism.

## 5. Conclusions

A large-scale Al-Cu-Mg aluminum alloy part was successfully achieved using AFSD in this study. A comprehensive investigation of the microstructure and corrosion behavior of the feedstock AFSD sample was conducted. The conclusions are as follows:

(1) The typical rolled microstructure of the studied Al-Cu-Mg alloy was refined by the AFSD process. The grain size of the AFSD-ed sample was refined to below 4 μm compared to an average grain size of 35.84 μm in the as-received rod. This is attributed to the dynamic recrystallization that occurred during AFSD.

(2) The AFSD-ed Al-Cu-Mg alloy exhibits improved corrosion resistance under the adopted electrochemical testing conditions, as evidenced by a higher corrosion potential and a lower corrosion current density. Specifically, the corrosion potential (*E_corr_*) of the Al-Cu-Mg alloy increased from −684.33 mV (vs. SCE) to −675.29 mV (vs. SCE), while the corrosion current density (*I_corr_*) decreased from 4.33 μA/cm^2^ to 1.45 μA/cm^2^ after AFSD.

(3) Through analysis of electrochemical impedance spectroscopy (EIS), the Al-Cu-Mg alloy after AFSD exhibits an increase in both capacitive arc diameter and impedance modulus, indicating effectively improved corrosion resistance. This enhancement may be related to the microstructural homogenization and refinement of second-phase particles induced by AFSD, but further quantitative characterization is needed to confirm this mechanism.

## Figures and Tables

**Table 1:** Chemical composition of Al-Cu-Mg aluminum alloy (wt.%).

Element	Cu	Mg	Mn	Ti	V	Si	Fe	Al
Content	4.64	0.95	0.01	0.01	0.01	0.05	0.12	Bal.

**Table 2:** The corrosion test results of the as-received and AFSD-ed Al-Cu-Mg alloy.

Materials	*E_ocp_* (mV)	*E_corr_* (mV)	*I_corr_* (μA/cm^2^)	*R_p_* (Ω·cm^2^)
As-received sample	−720.0 ± 1.0	−684.3 ± 0.9	4.33 ± 1.30	(7.60 ± 2.29) × 10^2^
AFSD-ed sample	−670.0 ± 1.0	−675.3 ± 7.8	1.45 ± 0.04	(1.59 ± 0.54) × 10^3^

**Table 3:** The fitting parameters from EIS of the as-received and AFSD-ed Al-Cu-Mg alloy.

Fitting Parameters	As-Received Sample	AFSD-Ed Sample
χ^2^	5.82 × 10^−3^	3.13 × 10^−3^
*R_S_*, Ω·cm^2^	6.271	5.236
*CPE*_1_ (*Q*), S·s^n^·cm^−2^	8.969 × 10^−5^	2.364 × 10^−6^
*n_1_*	0.9309	0.8979
*R_t_*, Ω·cm^2^	2.588 × 10^3^	1.843 × 10^3^
*CPE*_2_ (*Q*), S·s^n^·cm^−2^	5.023 × 10^−4^	5.911 × 10^−4^
*n*_2_	0.9604	0.9558
*R*_2_, Ω·cm^2^	5.976 × 10^3^	1.227 × 10^4^

## Data Availability

The original contributions presented in this study are included in the article. Further inquiries can be directed to the corresponding author.

## References

[1] Xie K., Nie J., Liu C., Cha W., Wu G., Liu X., Liu S. (2023). A Novel Al–Cu Composite with Ultra-High Strength at 350 °C via Dual-Phase Particle Reinforced Submicron-Structure. Adv. Sci..

[2] De D., De Postgrado E., De D., De A., Al-Cu-Mg A.R. UNIVERSIDAD AUTÓNOMA DE NUEVO LEÓN FACULTAD DE INGENIERÍA MECÁNICA Y ELÉCTRICA POR. https://d1wqtxts1xzle7.cloudfront.net/48841025/37394_8479_2-matrices_y_vectores-libre.pdf?1473887377=&response-content-disposition=inline%3B+filename%3DUNIVERSIDAD_AUTONOMA_DE_NUEVO_LEON_FACUL.pdf&Expires=1793067727&Signature=GqC8ho4qklgccta~BjLqSEdzvwDmw3EY7dPPia29grwU4HiGNJEi~XDULonNCOwadV4oita2SvEmANeOcnJ32FQiqdrn4cj3szUJjoFsKjOyksjbiJWqtc51PxWxvmdc-aVrVi7ActxfA4fkKhT8i-dvgDB2QaJGJFIuio7Q6WT-3~vGrxmM8z~kVqunt44MRpS~lOGaJC4gmZO2LIpuQQIDH9dZnXYLvKsRpDPM9B5npOdFaSMJqjxXO5aMWFdnFewMY-cVdBufpXDZR-VWKapue5Av474d80wVIrGgMD18uxa3WM7oawPeQDAsnCIx0zgAZVTVnNt1TblWWc9ppQ__&Key-Pair-Id=APKAJLOHF5GGSLRBV4ZA.

[3] Czerwinski F. (2020). Thermal stability of aluminum alloys. Materials.

[4] Cui L. (2024). Lightweight Al-Based Entropy Alloys for Elevated-Temperature Applications. Ph.D. Thesis.

[5] Kumar Makineni S., Sugathan S., Meher S., Banerjee R., Bhattacharya S., Kumar S., Chattopadhyay K. (2017). Enhancing elevated temperature strength of copper containing aluminium alloys by forming L12 Al3Zr precipitates and nucleating θ″ precipitates on them. Sci. Rep..

[6] Mishra R.S., Ma Z.Y. (2005). Friction stir welding and processing. Mater. Sci. Eng. R Rep..

[7] Gao P., Zhang Y., Mehta K.P. (2021). Metallurgical and Mechanical Properties of Al–Cu Joint by Friction Stir Spot Welding and Modified Friction Stir Clinching. Korean Inst. Met. Mater..

[8] Jeong W., Yang J., Choi J.P., Hwang J.Y., Kim Y.W., Park S.J., Choi J.W., Heogh W., Lee H., Park J. (2025). Al-based functionally graded super-intermetallic compounds for the turbine blade of a high-performance jet engine. Adv. Compos. Hybrid Mater..

[9] Dong X., Zhou M., Geng Y., Han Y., Lei Z., Chen G., Shi Q. (2024). Recent Advances in Additive Friction Stir Deposition: A Critical Review. Materials.

[10] Shao J., Samaei A., Xue T., Xie X., Guo S., Cao J., MacDonald E., Gan Z. (2023). Additive friction stir deposition of metallic materials: Process, structure and properties. Mater. Des..

[11] Ghadimi H., Ding H., Emanet S., Talachian M., Cox C., Eller M., Guo S. (2023). Hardness Distribution of Al2050 Parts Fabricated Using Additive Friction Stir Deposition. Materials.

[12] Feng X., Zhang M., Jiang T., Xie Y., Sun Z., Li W. (2024). Additive friction stir deposition of an Al-Cu-Mg-Mg alloy: Microstructure evolution and mechanical properties. Mater. Charact..

[13] Griffiths R.J., Garcia D., Song J., Vasudevan V.K., Steiner M.A., Cai W., Hang Z.Y. (2021). Solid-state additive manufacturing of aluminum and copper using additive friction stir deposition: Process-microstructure linkages. Materialia.

[14] Wang Z., Qian H., Chang W., Yu Z., Qiao Q., Zhou M., Guo D., Zhang D., Kwok C.T., Tam L.M. (2024). Microstructure and localized corrosion properties of 2219 aluminum alloy manufactured by additive friction stir deposition. Corros. Sci..

[15] Liu W., Zhang S., Li N., Zheng J., An S., Xing Y. (2012). Dealloying Behavior of Dual-Phase Al 15 at.% Cu Alloy in an Acidic Solution. http://www.electrochemsci.org.

[16] Dai W., Zhang C., Zhao L., Li C. (2022). Effects of Cu content in Al-Cu-Mg alloys on microstructure, adhesive strength, and corrosion resistance of thick micro-arc oxidation coatings. Mater. Today Commun..

[17] Román A.S., Méndez C.M., Ares A.E. (2014). Corrosion resistance of Al-Cu-Mg alloys in function of the microstructure. Mater. Sci. Forum.

[18] Barker M. (2010). Localized corrosion of a 2219 aluminium alloy exposed to a 3.5% NaCl solution. Corros. Sci..

[19] Ales S.K., Wang L. (2017). Effects of Friction Stir Welding on Corrosion Behaviors of AA2024-T4 Aluminum Alloy. MATEC Web Conf..

[20] Kung T.M., Liu C.P., Chang S.C., Chen K.W., Wang Y.L. (2010). Effect of Cu-Ion Concentration in Concentrated H3PO4 Electrolyte on Cu Electrochemical Mechanical Planarization. J. Electrochem. Soc..

[21] Ikeuba A.I., Njoku C.N., Ekerenam O.O., Njoku D.I., Udoh I.I., Daniel E.F., Uzoma P.C., Etim I.I.N., Okonkwo B.O. (2024). A review of the electrochemical and galvanic corrosion behavior of important intermetallic compounds in the context of aluminum alloys. R. Soc. Chem..

[22] Xie Y., Meng X., Wang F., Jiang Y., Ma X., Wan L., Huang Y. (2021). Insight on corrosion behavior of friction stir welded AA2219/AA2195 joints in astronautical engineering. Corros. Sci..

[23] Zhang B., Wang J., Wu B., Oguzie E.E., Luo K., Ma X.L. (2016). Direct observation of atomic-scale origins of local dissolution in Al-Cu-Mg-Mg alloys. Sci. Rep..

[24] Gharbi O. In-Situ Investigation of Elemental Corrosion Reactions During the Surface Treatment of Al-Cu-Mg and Al-Cu-Mg-Li Alloys. https://theses.hal.science/tel-01535612v1.

[25] Peng Y., Huang B., Zhong Y., Su C., Tao Z., Rong X., Li Z., Tang H. (2023). Electrochemical corrosion behavior of 6061 Al alloy under high rotating speed submerged friction stir processing. Corros. Sci..

[26] Zavari S., Bagheri E., Ding H., Adibi N., Eller M., Cox C., Guo S. (2025). The influence of additive friction stir deposition process on mechanical properties, corrosion resistance, and electrical conductivity of Al5086-H32 alloy. Prog. Addit. Manuf..

[27] Qiao Q., Chen X., Lam W.I., Lin Y., Wang H., Qian H., Li Z., Guo D., Zhang D., Kwok C.T. (2025). Hybrid heat-source solid-state additive manufacturing of 5A06 deposition with favorable mechanical and electrochemical performance. NPJ Mater. Degrad..

[28] Li Z., Guo D., Qian H., Zhang D., Wu Z., Qiao Q., Tam L.M., Kwok C.T. (2026). Corrosion behavior of AA2024 aluminum alloy manufactured by solid state additive manufacturing-additive friction stir deposition. Corros. Sci..

[29] Zhou Z., Shen Y., Lyu W., Xiong W., He Z., Lin Y., Zhou Z., Guo X. (2025). Interface stair-like design and repair performance of Al-Zn-Mg-Cu aluminum alloy based on additive friction stir deposition. J. Mater. Process. Technol..

[30] Babu A.P., Choudhary S., Griffith J.C., Huang A., Birbilis N. (2021). On the corrosion of a high solute Al-Zn-Mg alloy produced by laser powder bed fusion. Corros. Sci..

